# Generation of interconnected vesicles in a liposomal cell model

**DOI:** 10.1038/s41598-020-70562-5

**Published:** 2020-08-20

**Authors:** Baharan Ali Doosti, Daniel Fjällborg, Kiryl Kustanovich, Aldo Jesorka, Ann-Sofie Cans, Tatsiana Lobovkina

**Affiliations:** Department of Chemistry and Chemical Engineering, Chalmers University of Technology, Kemivägen 9, 412 96 Göteborg, Sweden

**Keywords:** Imaging studies, Bioinspired materials, Phospholipids, Biophysical chemistry, Chemical tools, Membranes, Synthetic biology

## Abstract

We introduce an experimental method based upon a glass micropipette microinjection technique for generating a multitude of interconnected vesicles (IVs) in the interior of a single giant unilamellar phospholipid vesicle (GUV) serving as a cell model system. The GUV membrane, consisting of a mixture of soybean polar lipid extract and anionic phosphatidylserine, is adhered to a multilamellar lipid vesicle that functions as a lipid reservoir. Continuous IV formation was achieved by bringing a micropipette in direct contact with the outer GUV surface and subjecting it to a localized stream of a Ca^2+^ solution from the micropipette tip. IVs are rapidly and sequentially generated and inserted into the GUV interior and encapsulate portions of the micropipette fluid content. The IVs remain connected to the GUV membrane and are interlinked by short lipid nanotubes and resemble beads on a string. The vesicle chain-growth from the GUV membrane is maintained for as long as there is the supply of membrane material and Ca^2+^ solution, and the size of the individual IVs is controlled by the diameter of the micropipette tip. We also demonstrate that the IVs can be co-loaded with high concentrations of neurotransmitter and protein molecules and displaying a steep calcium ion concentration gradient across the membrane. These characteristics are analogous to native secretory vesicles and could, therefore, serve as a model system for studying secretory mechanisms in biological systems.

## Introduction

Giant unilamellar vesicles (GUVs) are versatile model structures for studying the properties and behavior of cell membranes, owing to their similarity in composition, size and key physical properties. The development of such model systems is of significant interest, as the cell membrane is heavily involved in vital cellular processes, such as cell division, migration, endocytosis, and exocytosis^1^. Similar to the processes observed in biological cells, reshaping of GUVs can occur in response to external stimuli. For example, GUV shrinkage, budding, or formation of tubular extensions can result from changes in osmolarity or temperature, or through exposure to oligonucleotides, nanoparticles or proteins such as BAR domains or clathrin^2–8^. To adequately mimic cell processes, it is of utmost importance that cell model systems include key molecular components that would be present in a living cell. This could, for example, include the presence of F-actin, PI(4,5)P_2_ or calcium ions at physiologically relevant concentrations, which may be modulated to mimic cellular oscillations^9^. Such oscillations can be achieved in a GUV-based cell model system by exposing a small part of the GUV surface to external stimuli, using a microinjection technique. Recent studies with GUV-based cell models have focused on investigating the effect of localized variations in concentrations of DNA, hydrogen ions, and calcium ions in cell membrane dynamics^9–15^. Specifically, it was revealed that the binding of calcium ions to lipid membranes leads to a tighter packing of lipid head groups, an increase in ordering of the lipid hydrocarbon chains, and an increase of membrane rigidity and tension^16–19^. Our previous study has shown that contactless localized exposure of a GUV membrane to a calcium ion gradient, results in membrane remodeling and the formation of tubular protrusions at the site of calcium ion application^20^.

In the present study, microinjection of Ca^2+^ solution via a glass micropipette, placed in direct contact to the surface of a GUV, establishes locally a high calcium concentration region, which is confined by the opening of the micropipette tip. Following calcium ion application, we observed formation of strings of nanotube-interconnected vesicles, formed at the injection site upon consumption of membrane material from the host GUV. We refer to these vesicles as interconnected vesicles (IVs), whose contents are fully separated from the original vesicle contents, and are defined by the solution filling micropipette. Furthermore, by supplementing the calcium ion solution with additional solutes, IVs can be loaded with additional contents such as proteins or neurotransmitters in high concentrations. This, therefore, makes the functionalized IVs suitable model systems for studying transmembrane transport processes, for example endocytic vesicle formation, compartmentalization, and the dynamics of intracellular membrane structures.

**Figure 1:**
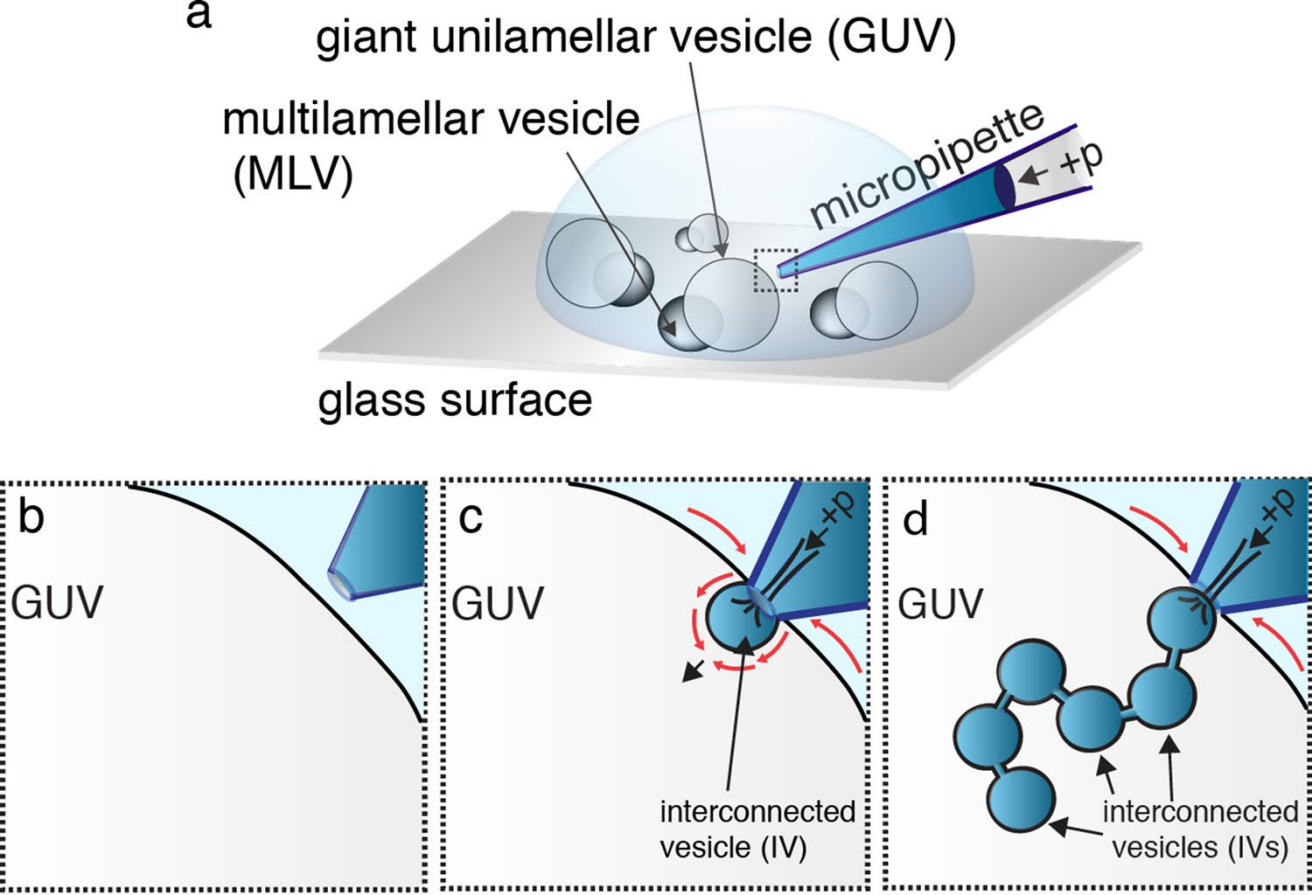
Schematic illustration of the sequential steps of micromanipulation and microinjection required for IV formation. (**a**) Placement of the micropipette tip into the bulk sample solution containing surface-attached GUV-MLVs. (**b**) Positioning of the micropipette tip near the GUV surface. (**c**) A positive pressure (+ p) is applied to the micropipette while putting the pipette into direct contact with the GUV membrane, which results in a flow of calcium ion solution and inflation of the first IV. (**d**) Applying a continuous injection of calcium ion solution results in synthesis of a multitude of IVs, which are filling the interior of the GUV. The illustrations were drawn in Adobe Illustrator CS6.

The requirements for IV generation include a surface-adhered GUV with a membrane reservoir (multilamellar vesicle, MLV) connected to it, calcium ions in relevant concentrations, and a microinjection system. The MLV, or membrane onion-shell layered vesicle, with a continuous connection to the GUV, serves as a source of lipids for IV formation. In the presence of a local membrane tension increase, as it occurs at the site of calcium dispensing at the GUV surface, lipid material is transported via a Marangoni process from the MLV reservoir towards the site of high tension^21–23^. In our study, the GUV–MLV assembly was prepared using a dehydration-rehydration method. The lipid mixture consisted of soybean polar lipid extract (SPE; 80 wt.%), and 1,2-dioleoyl-sn-glycero-3-phospho-L-serine (DOPS; 20 wt.%)^19^, unless otherwise stated. GUV-MLV assemblies with a net negatively charged surface were successfully formed in HEPES buffer solution (10 mM; pH 7.4) and adhered onto a glass coverslip by self-adsorption, rendering them suitable for micromanipulation during experiments (Fig. 1a). For further details on materials and methods including the full GUV-MLV preparation procedure, consult the methods section.

**Figure 2:**
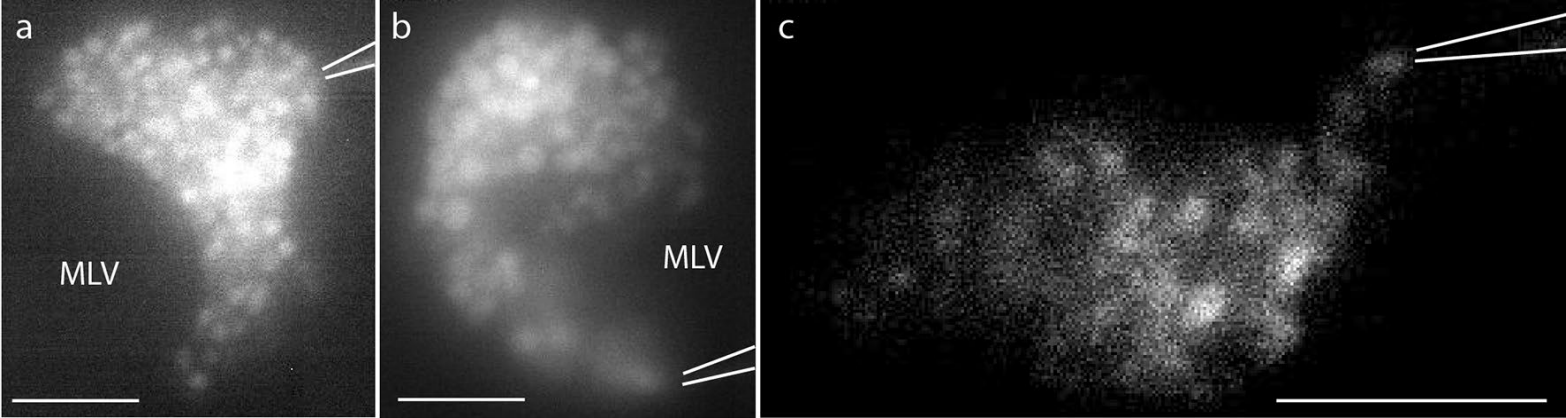
Fluorescence microscopy images illustrating calcium-ion assisted formation of IVs. (**a**) Formation of the IVs upon a 25 s injection of calcium ion solution, supplemented with 10 µM Alexa-488 for visualization when placing the micropipette tip in contact with the GUV surface. (**b**) IVs co-loaded with streptavidin − 488 (10 µM). Injection time is 12 s. (**c**) IVs co-loaded with a glutamate solution (10 µM with Alexa 488 NHS ester co-solute). Injection time is 3 s. The images were brightness/contrast-enhanced for improved visualization. The white lines outline the position of the micropipette tip in the experiments. The images were prepared using the NIH ImageJ software, VirtualDub 1.10.4, and Adobe Illustrator CS6. The scale bars represent 5 µm.

Following the formation of the GUV–MLV assemblies, a glass micropipette with an open tip (Fig. S1A; Table S1), filled with a CaCl_2_ solution (5 mM) in HEPES buffer (10 mM), supplemented with Alexa-488 (10 µM) for visualization purposes, was positioned 5 µm away from the GUV membrane. At this distance, a positive injection pressure of 20–25 hPa was applied to the micropipette, resulting in a continuous net flow of solution and release of calcium ions from the pipette tip. The micropipette was then promptly brought into direct contact with the GUV surface. Maintaining the continuous flow of the calcium ion solution from the pipette resulted in the formation of IVs, which were produced one-by-one, growing into the GUV interior (Figs. 1 and 2). IV formation was terminated either by the exhaustion of the lipid reservoir, i.e., depletion of lipid material from the MLV (Movie S1) or by discontinuing the injection flow from the tip. When turning the calcium ion supply off, the IVs remained stable during the period of at least 30 min, retaining the original size and abundance (Fig. S2).

In addition to the lipid reservoir (i.e. MLV), the generation of IVs required both a supply of calcium ions in the microinjection solution as well as a direct physical contact of the micropipette with the GUV surface. We observed that in the absence of calcium ions, IVs were not able to form. Our earlier work has revealed that GUVs exposed to a continuous contactless injection of calcium ion solution at a distance of 3–5 µm away from the GUV surface resulted in the formation of tubular protrusions instead of IV formation^20^. We found that calcium ions that bind to the net negatively charged lipid membrane at the exposed side of the GUV neutralize the negative surface charges. This results in a mismatch in surface charge density across the lipid bilayer and induces a membrane spontaneous curvature sufficient to bend the bilayer away from the ion source^20^. We suggest that a similar mechanism of membrane bending contributes to the formation of IVs, i.e. the spontaneous curvature induced upon application of calcium ions at the outer surface of the GUV leads to inward membrane bending, allowing IVs to form.

### The IVs are connected to each other like beads on a string

**Figure 3:**
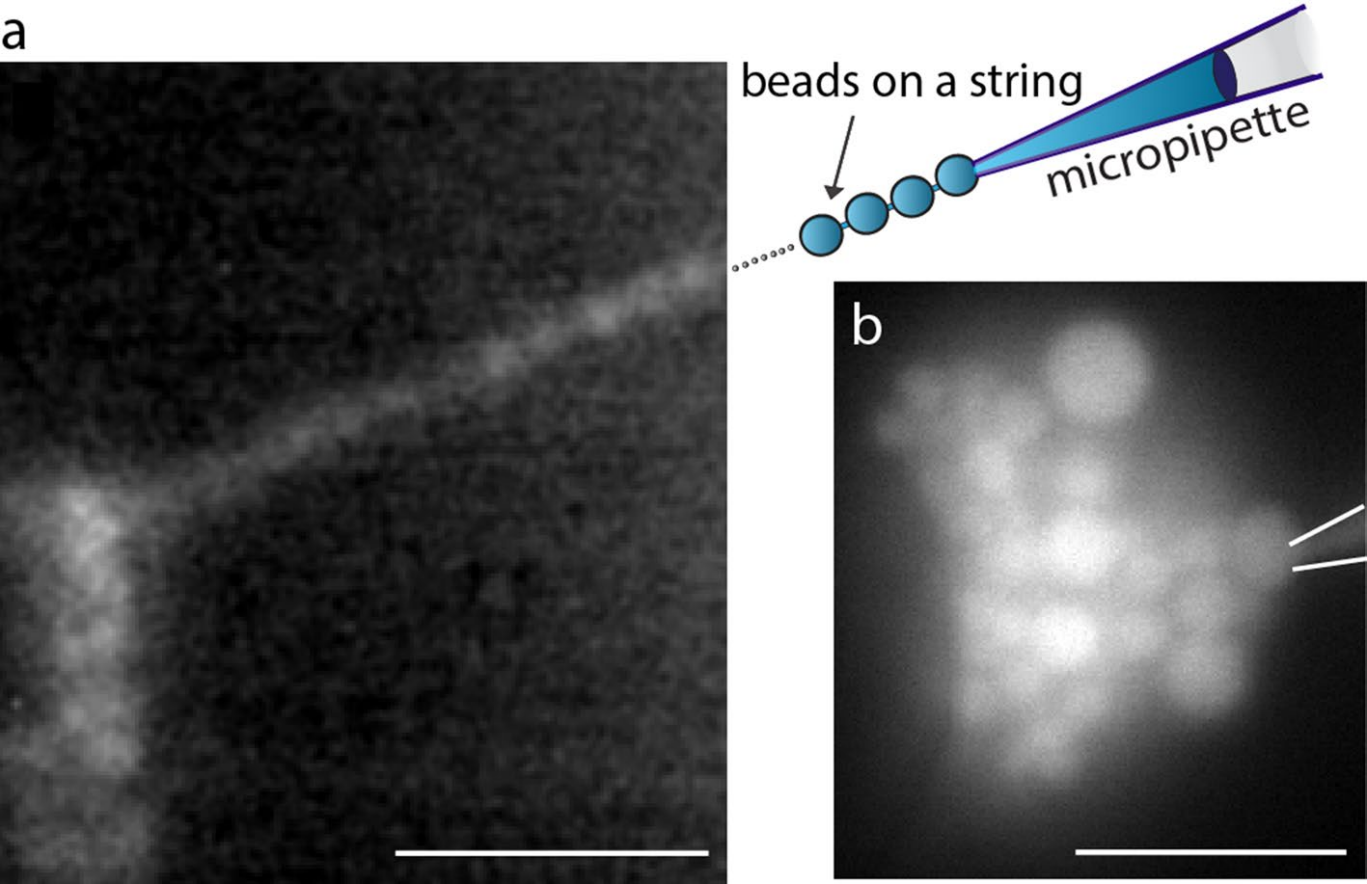
Fluorescence microscopy images demonstrating the formation of IVs and their connectivity. (**a**) By pulling the micropipette (0.3 µm in diameter) away from the GUV, the IVs attached to the micropipette tip were pulled out of the GUV interior and displayed the vesicle connectivity of “beads on a string”. (**b**) A 5 s injection of calcium ion solution using a micropipette with an inner tip diameter of 2 µm resulted in the formation of polydisperse IVs. The images were prepared using the NIH ImageJ software, VirtualDub 1.10.4, and Adobe Illustrator CS6. The scale bars represent 5 µm.

When continuously produced one-by-one, the IVs can be generated at large numbers and fill a large portion of the interior of the GUV. To address the connectivity of IVs, we performed an experiment in which a membrane-soluble fluorescent dye, FM1-43, was added to the bulk solution after the IVs were formed. The micropipette was retracted from the GUV membrane after the IV formation and was not in contact with the GUV when FM1-43 was added to the bulk solution. Due to its amphipathic properties, this fluorophore stains solely the bilayer leaflet exposed to the medium containing the dye^24^. Upon entering the lipid membrane, FM1-43 dye fluoresces brightly, whereas in aqueous solution negligible fluorescence is detected. Exposure of a GUV containing newly synthesized IVs to FM1-43 (final concentration 0.02 mg/mL) resulted in the bright fluorescence of both the GUV and IV membranes (see Fig. S4). The observed diffusion of the dye from the bulk solution into the membrane of the IVs indicates that IVs are indeed interconnected via a narrow membrane passage, as well as to a point source at the GUV membrane. Furthermore, the connectivity could be directly verified by reversing the direction of flow in the micropipette after the formation of the IVs. In this experiment, a portion of the GUV membrane at the injection site was aspirated into the pipette allowing a tight attachment of the GUV membrane to a micropipette. By pulling the pipette tip away from the GUV, we were able to visualize a string of connected IVs (Fig. 3a).

### Negligible leakage from the pipette tip upon formation of IVs

The interior of the IVs is defined by the content of the micropipette. To investigate whether the solution from the micropipette was delivered into the IVs directly, without detectable leakage, we added the pre-fluorescent calcium ion indicator Fluo-3 to the GUV-exterior solution. The Fluo-3 fluorescence intensity is 40 times greater in the presence of calcium ions, compared to a calcium ion free-solution. The Fluo-3 was pipetted into the bulk solution to a final concentration of 0.02 mg/mL. With the micropipette in contact with the GUV membrane during the microinjection, Fluo-3 fluorescence was indistinguishable from the background level (Fig. S3A). A control with the micropipette tip at the distance of ~ 5 µm from the GUVs resulted in bright fluorescence at the injection site (Fig. S3B).

### Influence of micropipette tip size and membrane composition on the size of IVs

In order to investigate the possibility to control the IV diameter size, we first altered the micropipette tip size. When using a tip diameter of 0.3 µm resulted in monodispersed IVs with an average diameter of 0.8 µm ± 0.2 µm, whereas a 2 µm tip diameter (Fig. S1B; Table S1) resulted in IV formation with an uneven size distribution and with a diameter ranging from 0.7 to 1.7 µm (Fig. 3b). Next, to address the influence of membrane composition on the process of IV formation, we incorporated cholesterol into the GUV membrane. Cholesterol, which is an abundant sterol in organelle and cellular plasma membranes (~ 20–40% of the lipid content^25,26^), is known for influencing membrane rigidity as well as modulating fluidity of cellular and lipid membranes upon temperature variations^27,28^. Here, we investigated how varying the GUV membrane cholesterol content from 0, to 10 and 20 wt.% affects the IV size. The inclusion of 10 or 20% cholesterol into the GUV membrane resulted in the formation of IVs that appeared slightly smaller, with the average diameter of 0.7 µm ± 0.1 µm compared to IVs formed using the cholesterol-free membrane. The overall effect of cholesterol appears to be minute, regardless of the large concentration variation of cholesterol within the membrane.

### IVs loaded with glutamate can be used as a simple model system of secretory vesicles

The generated IVs (Fig. 2c) resemble intracellular organelles, comparable to the secretory vesicles that are involved in the exocytosis process by secretory cells during cellular communication. In addition to a very high concentration of neurotransmitters, ATP, ascorbate, magnesium ions, and calcium ions, which are stored inside secretory vesicles like the synaptic vesicles found in neurons, vesicles such as the large “dense core vesicles” also contain a condensed protein matrix, which reduce the osmolarity of the internal organelle solution and prevent vesicle rupture^29–34^. Therefore, in order to create a secretory vesicle mimic, an imitation of both synaptic vesicles and the dense core secretory granule content was formulated. In addition to the calcium ions, the IVs were loaded with a protein or a neurotransmitter solution. As a model of protein, we chose fluorescently labeled streptavidin (streptavidin-488, 10 µM) as this protein does not bind to the GUV membrane, and therefore was not anticipated to affect IV formation. As a neurotransmitter, we chose glutamate (in concentrations of 10, and 100 µM) due to its abundance in the brain and central nervous system. To load the IVs with streptavidin or glutamate, the micropipette was filled with a calcium ion solution (5 mM) supplemented with either fluorescently labeled streptavidin (with Alexa-488) or with glutamate and Alexa-488 dye. IVs of similar size and appearance to those synthesized with solely calcium ion solution were formed, as shown in Fig. 2b,c. When exposing the GUV surface to a local flow of a calcium-free streptavidin or glutamate solution, no formation of IVs was observed.

### Discussion on the nanotube interconnected IVs

Cylindrical lipid nanotubes under tension are known to be subject to the spontaneous pearling, reminiscent of the Plateau-Rayleigh instability^35,36^. The phenomenon we observe, where IVs are nanotube-interconnected, is most likely different, as it does not describe a transformation of a fluid thread under the influence of an external source of tension. It rather resembles the pearling observed in biological cells after destruction of the actin cortex^37,38^, and can also be related to spontaneous curvature^20,39,40^. Calcium interacts strongly with negatively charged components of a lipid bilayer and is known to be partitioned into the membrane, even at high µM concentrations^6^. This leads to both an imbalance between the inner and outer monolayer leaflet of the affected membrane regions, causing segregation of lipids, as well as membrane budding^6,17,41^. The high calcium concentration in the interior of the vesicle chain might thus be responsible for the stabilization of the nanoscale interconnections. However, the limiting of vesicle growth in the formation process, leading to a string of nearly homogenously sized vesicles, cannot be easily explained by the influence of calcium and requires further elucidation.

## Summary

In this study, we present a method to generate GUV-internalized IVs based upon a microinjection technique. The IVs are generated by applying a calcium ion-containing solution through a glass micropipette that is positioned in direct contact with the surface of the GUV. The IVs are produced in a bead-on-a-string fashion, maintaining a continuous membrane connection to the GUV at the injection site of the calcium-containing solution. The interior of the IVs is filled with the solution from the micropipette, which allows for the loading of the IVs with multi-component solutions at physiologically relevant concentrations. Specifically, the IVs loaded with a high content of protein or neurotransmitter solution show a great promise to serve as a model system for studies of secretory vesicles. The size of the generated IVs can to some degree be controlled by varying the diameter of the micropipette tip. Potential applications of this technique include mimicking cellular processes relating to compartmentalization, and cell functions such as exocytosis and endocytosis, that are based on fusion and retrieval of intracellular vesicles under asymmetric calcium concentration conditions. The individual protein-loaded IVs also bear potential in time-delayed drug delivery applications due to the high concentration of the entrapped molecules in combination with stable encapsulation. Future investigations could involve disconnection of the IVs from each other to generate solitary vesicles and reversal of the IV encapsulation for release of the internalized contents.

## Methods

### Chemicals

Soybean Polar Lipid Extract (SPE) and 1,2-dioleoyl-sn-glycero-3-phospho-L-serine (DOPS) was purchased from Avanti Polar Lipids, Inc. (Alabaster, USA). Chloroform, PBS tablets, l-glutamic acid, and piperazine-1-ethanesulfonic acid (HEPES) solution (1 M) were obtained from Sigma-Aldrich (Missouri, USA). CaCl_2_ was purchased from KEBO lab (Sweden). Streptavidin Alexa Fluor 488 conjugate, Alexa Fluor 488 NHS Ester (Succinimidyl Ester), FM 1–43 Dye (*N*-(3-Triethylammoniumpropyl)-4-(4-(Dibutylamino) Styryl) Pyridinium Dibromide), and Fluo-3 AM calcium indicator were purchased from Thermo Fisher Scientific (Sweden).

### Formation of GUV–MLV complexes

The GUV–MLV (giant unilamellar—multilamellar vesicles) complexes were formed using a dehydration–rehydration protocol^42^. In short, SPE and DOPS lipids, were mixed in chloroform at 80/20 wt.% ratio in a rotary evaporation flask, or with additional 10 or 20 wt.% cholesterol to reach a final lipid concentration of 1 mg/mL. The SPE composition consists of the following phospholipids: PC (phosphatidylcholine) 45.7%, PE (phosphatidylethanolamine) 22.1%, PI (phosphatidylinositol) 18.4%, PA (phosphatidic acid) 6.9%, and a mixture of other lipids 6.9%. The lipids in chloroform solvent were evaporated in a rotary evaporator for 3 h to form a dry lipid film. Thereafter, the film was rehydrated by adding 0.6 mL of PBS buffer (pH 7.8) supplemented with 1 wt.% glycerol, and was incubated over night at 4 °C. This was followed by 1 min ultrasonication to form small vesicles (SV) in suspension. The SV suspension was stored at − 18 °C until use. For experiments, a droplet (5 µL) of the SV suspension was placed on a glass cover slip and placed inside a vacuum desiccator for 20 min for dehydration. Then, 50 μL of 10 mM HEPES buffer (pH 7.4) was added to rehydrate the lipid film during a 5 min incubation period. The lipid solution was transferred onto a new glass cover slip containing 300 µL of HEPES buffer solution. The GUV–MLVs were finally allowed to adsorb to the new cover slip surface for 30 min.

### Microscopy imaging

The images were obtained using an inverted fluorescence microscopy system (Leica DM IRB, Wetzlar, Germany), equipped with a 100 × oil immersion objective (Leica, 100 × , 1.4 NA, oil immersion). A 488 nm laser line (Cobolt MLD-488 nm) enabled excitation of the Alexa-488 fluorophore and visualization of the dye FM1-43. A camera (Prosilica Ex 1920, Allied Vision Technologies GmbH, Thuringia, Germany) and a custom-made script in Labview 2009 (National Instruments) was used to collect the data. Fluorescence images and movies were edited and improved using NIH ImageJ software and VirtualDub 1.10.4. The fluorescence microscopy images and movies were enhanced using the operations Gaussian blur, Brightness and Contrast in the NIH ImageJ software. In addition, to better visualize the calcium ion flow into the buffer solution, the LUT (spectrum) was added in Fig. S4.

### IV diameter measurements

The diameters of the IVs were measured using raw single image frames from movie recordings during IV formation. Here, four crossline segments were defined to determine the average diameter of single IVs. To account for variation in depth profile when recording IVs during formation inside the GUV, an average IV size (+ /- S.D) was determined from a series of consecutive movie images (n (IVs) = 4–10; n (GUVs) = 16). The intensity values were obtained using a Plot Profile function in the NIH ImageJ software.

## Supplementary information

Supplementary file1

Supplementary file2

## References

[1] Walde P, Cosentino K, Engel H, Stano P (2010). Giant vesicles: preparations and applications. ChemBioChem.

[2] Bahrami A (2014). Wrapping of nanoparticles by membranes. Adv. Colloid Interface Sci..

[3] Zimmerberg J, McLaughlin S (2004). Membrane curvature: How BAR domains bend bilayers. Curr. Biol..

[4] Oglęcka K, Rangamani P, Liedberg B, Kraut R, Parikh A (2014). Oscillatory phase separation in giant lipid vesicles induced by transmembrane osmotic differentials. eLife..

[5] Saletti D (2017). The Matrix protein M1 from influenza C virus induces tubular membrane invaginations in an in vitro cell membrane model. Sci. Rep..

[6] Melcrová A (2016). The complex nature of calcium cation interactions with phospholipid bilayers. Sci. Rep..

[7] Farsad K, Camilli P (2003). Mechanisms of membrane deformation. Curr. Opin. Cell Biol..

[8] Gözen I (2013). Thermal migration of molecular lipid films as a contactless fabrication strategy for lipid nanotube networks. Lab Chip..

[9] Wollman R, Meyer T (2012). Coordinated oscillations in cortical actin and Ca2+ correlate with cycles of vesicle secretion. Nat. Cell Biol..

[10] Khalifat N, Puff N, Bonneau S, Fournier J, Angelova M (2008). Membrane deformation under local pH gradient: Mimicking mitochondrial cristae dynamics. Biophys. J..

[11] Angelova M, Hristova N, Tsoneva I (1999). DNA-induced endocytosis upon local microinjection to giant unilamellar cationic vesicles. Eur. Biophys. J..

[12] Monteith G, McAndrew D, Faddy H, Roberts-Thomson S (2007). Calcium and cancer: targeting Ca2+ transport. Nat. Rev. Cancer..

[13] Berridge M, Bootman M, Lipp P (1998). Calcium—a life and death signal. Nature.

[14] Prevarskaya N, Skryma R, Shuba Y (2011). Calcium in tumour metastasis: new roles for known actors. Nat. Rev. Cancer..

[15] Leckband D, Helm C, Israelachvili J (1993). Role of calcium in the adhesion and fusion of bilayers. Biochemistry.

[16] Pedersen U, Leidy C, Westh P, Peters G (2006). The effect of calcium on the properties of charged phospholipid bilayers. Biochim. Biophys. Acta..

[17] Sinn C, Antonietti M, Dimova R (2006). Binding of calcium to phosphatidylcholine–phosphatidylserine membranes. Colloids Surf..

[18] Binder H, Zschörnig O (2002). The effect of metal cations on the phase behavior and hydration characteristics of phospholipid membranes. ChemPhysLipids..

[19] Boettcher J (2011). Atomic view of calcium-induced clustering of phosphatidylserine in mixed lipid bilayers. Biochemistry.

[20] Ali Doosti B (2017). Membrane tubulation in lipid vesicles triggered by the local application of calcium ions. Langmuir.

[21] Nasseri B, Florence A (2005). The relative flow of the walls of phospholipid tether bilayers. Int. J. Pharm..

[22] Karlsson R (2002). Moving-wall-driven flows in nanofluidic systems. Langmuir.

[23] Dommersnes P, Orwar O, Brochard-Wyart F, Joanny JF (2005). Marangoni transport in lipid nanotubes. EPL.

[24] Meyers J (2003). Lighting up the senses: FM1-43 loading of sensory cells through nonselective ion channels. J. Neurosci..

[25] Takamori S (2006). Molecular anatomy of a trafficking organelle. Cell.

[26] van Meer G, Voelker D, Feigenson G (2008). Membrane lipids: where they are and how they behave. Nat. Rev. Mol. Cell Biol..

[27] Goluszko P, Nowicki B (2005). Membrane cholesterol: a crucial molecule affecting interactions of microbial pathogens with mammalian cells. Infect. Immun..

[28] Rogasevskaia T, Churchward M, Coorssen J (2012). Anionic lipids in Ca2+-triggered fusion. Cell Calcium.

[29] Estévez-Herrera J (2016). ATP: The crucial component of secretory vesicles. Proc. Natl. Acad. Sci..

[30] Machado J, Camacho M, Alvarez J, Borges R (2009). On the role of intravesicular calcium in the motion and exocytosis of secretory organelles. Commun. Integr. Biol..

[31] Toll L, Howard B (1978). Role of Mg2+ion-activated ATPase and a pH gradient in the storage of catecholamines in synaptic vesicles. Biochemistry.

[32] Terland O, Flatmark T (1975). Ascorbate as a natural constituent of chromaffin granules from the bovine adrenal medulla. FEBS Lett..

[33] Camacho M, Machado J, Montesinos M, Criado M, Borges R (2006). Intragranular pH rapidly modulates exocytosis in adrenal chromaffin cells. J. Neurochem..

[34] Wightman R, Schroeder T, Finnegan J, Ciolkowski E, Pihel K (1995). Time course of release of catecholamines from individual vesicles during exocytosis at adrenal medullary cells. Biophys. J..

[35] Boedec G, Jaeger M, Leonetti M (2014). Pearling instability of a cylindrical vesicle. J. Fluid Mech..

[36] Bar-Ziv R, Moses E (1994). Instability and "pearling" states produced in tubular membranes by competition of curvature and tension. Phys. Rev. Lett..

[37] Bar-Ziv R, Tlusty T, Moses E, Safran SA, Bershadsky A (1999). Pearling in cells: A clue to understanding cell shape. PNAS.

[38] Heinrich D, Ecke M, Jasnin M, Engel U, Gerisch G (2014). Reversible membrane pearling in live cells upon destruction of the actin cortex. Biophys. J..

[39] Pezeshkian W, König M, Marrink SJ, Ipsen JH (2019). A multi-scale approach to membrane remodeling processes. Front. Mol. Biosci..

[40] Lipowsky R (2013). Spontaneous tubulation of membranes and vesicles reveals membrane tension generated by spontaneous curvature. Faraday Discuss..

[41] Drücker P, Pejic M, Galla H-J, Gerke V (2013). Lipid segregation and membrane budding induced by the peripheral membrane binding protein Annexin A2. J. Biol. Chem..

[42] Ali Doosti B, Cans A-S, Jeffries DM, Lobovkina T (2018). Membrane remodeling of giant vesicles in response to localized calcium ion gradients. J. Vis. Exp..

