# Supplementary material for: Generation of interconnected vesicles in a liposomal cell model
Source: Sci Rep. 2020 Aug 20;10:14040. doi: 10.1038/s41598-020-70562-5 (PMC7441142; doi:10.1038/s41598-020-70562-5)
Supplement: Supplementary file 2 — Supplementary file2 [file 41598_2020_70562_MOESM2_ESM.pdf]

Supplementary Information

Generation of Interconnected Vesicles in a Liposomal Cell Model

*Baharan Ali Doosti, Daniel Fjällborg, Kiryl Kustanovich, Aldo Jesorka, Ann-Sofie Cans, Tatsiana Lobovkina**

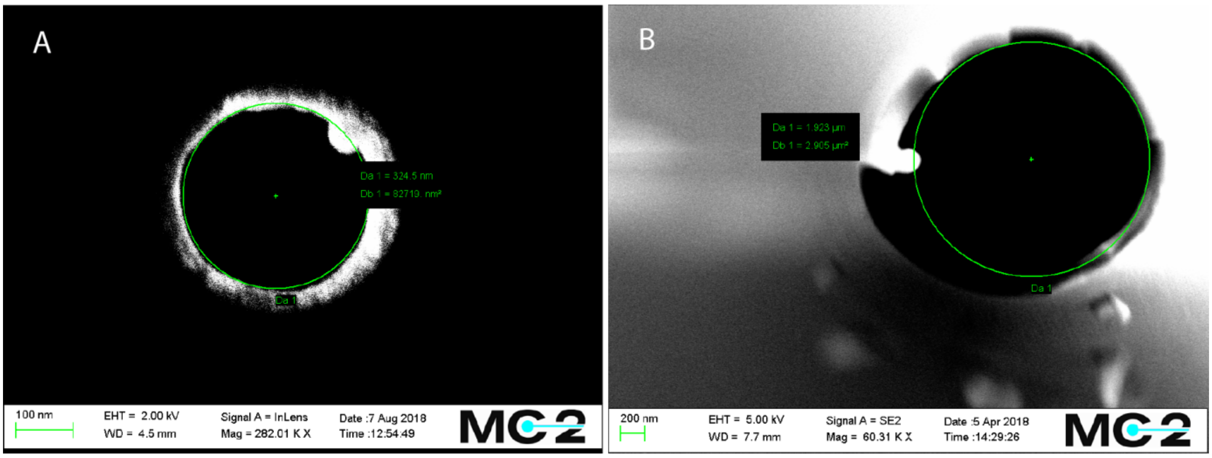

Figure S1. SEM images of the glass micropipette tip used in the experiments. A) The pipette tip with a 0.3 μm diameter. B) The pipette tip with a 2 μm diameter. The images were obtained using a Zeiss Supra 55 VP FEG-Scanning Electron Microscope (Carl Zeiss AG, Germany). Operation parameters are displayed in the panel legends (EHT: acceleration voltage; WD: working distance; Mag: Magnification).

Table S1. Parameters used in the glass micropipette puller (Sutter instrument Model P-1000) to generate the different micropipette tip sizes. The parameters for micropipette production; filament, heat, pull, velocity, delay time, pressure, and ramp that were selected to obtain tip sizes with diameters of 0.3 μm or 2 μm.

Tip opening diameter	Line	Filament	Heat	Pull	Velocity	Delay	Pressure	Ramp
**0.3 μm**	1	x	520	60	50	250	600	510
**2 μm**	1	x	472	100	40	240	500	462

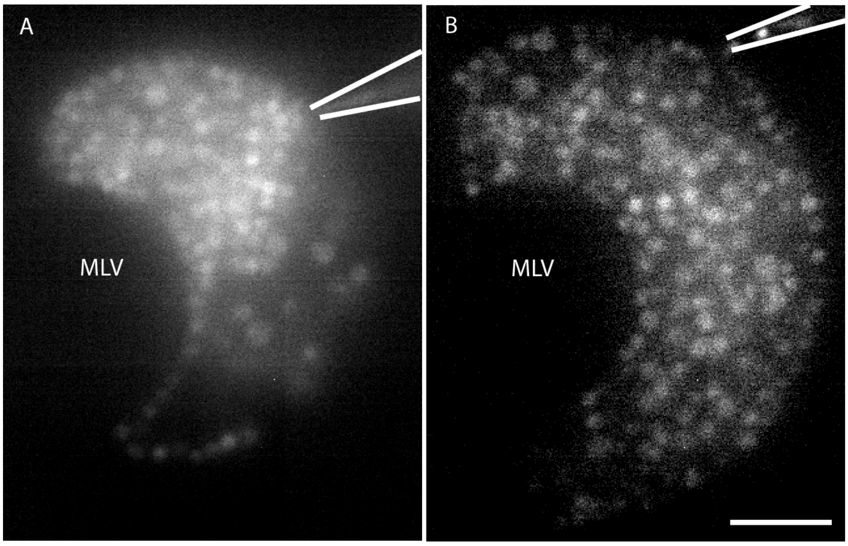

Figure S2. Fluorescence microscopy images demonstrating stability of the IVs. A) The IVs were formed inside the lumen of a GUV by injection of calcium ion solution containing Alexa-488 as fluorescent marker, using a 0.3 μm pipette tip and placed at the surface of a GUV composed of SPE/DOPS lipids. B) The IVs imaged after 31 min time period. The images were prepared using the NIH ImageJ software and Adobe Illustrator CS6. The scale bar represents 5 μm.

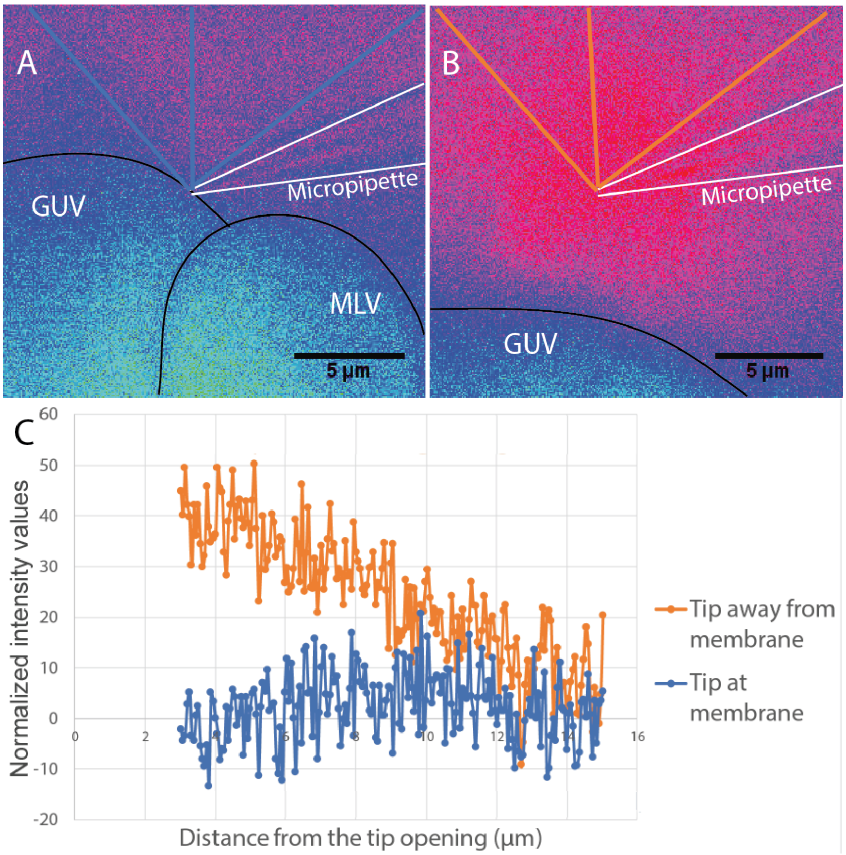

Figure S3. Fluorescence microscopy images showing that the inner content of the pipette tip is delivered into the IVs without leakage of calcium ions. A) Injection of calcium ion solution at the surface of the GUV in the presence of indicator dye Fluo-3 (0.02 mg/mL) in the bulk solution. B) Injection of the calcium ion solution ~ 5 μm away from the GUV surface. In each panel A and B, three 15 μm line segments were drawn in different directions from the pipette tip in order to obtain an average intensity values at the same distance from the pipette tip. C) Fluorescence intensity values (Ca^{2+}-Fluo-3 complex fluorescence) plotted against distance from the tip opening. Displayed are the averaged (± std) intensity values measured along the three line segments shown in A (blue) and B (orange). The intensity values were normalized, such that that zero value corresponds to an averaged background intensity measured across blue lines in the image S3A. The images were prepared using the NIH ImageJ software, Microsoft Excel, and Adobe Illustrator CS6.

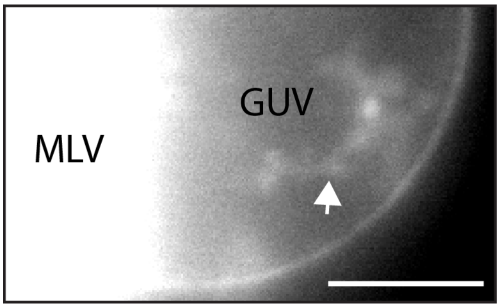

Figure S4. Fluorescent image showing IV formation as beads on a string. Addition of FM1-43 (0.02 mg/mL) to the bulk solution resulted in bright fluorescence of the GUV and the IV membranes, giving evidence that the IVs (pointed by the white arrow) are connected to each other and to the GUV membrane by an intact membrane. The image was prepared using the NIH ImageJ software and Adobe Illustrator CS6. The scale bar represents 5 μm.
